# Identification of progression-related genes and construction of prognostic model for chronic kidney disease by machine learning

**DOI:** 10.3389/fcell.2025.1627355

**Published:** 2025-08-15

**Authors:** Bingkun Zhou, Hu Zhou, Xiaodong Huang, Shijie Liu

**Affiliations:** ^1^ Department of Kidney Transplantation, The Third Affiliated Hospital of Sun Yat-sen University, Guangzhou, Guangdong, China; ^2^ Department of Medicine, Nephrology Division, The Third Affiliated Hospital, Sun Yat-sen University, Guangzhou, China; ^3^ Department of Urology, Sun Yat-sen Memorial Hospital, Sun Yat-sen University, Guangzhou, China

**Keywords:** chronic kidney disease, bioinformatics, transcriptomics, machine learning, predictive model

## Abstract

**Background:**

Early diagnosis and intervention for chronic kidney disease (CKD) can significantly improve patient’s quality of life and prognosis. Besides routine laboratory indicators and medical history, risk prediction models can predict CKD outcome. However, there is currently a lack of CKD prognostic prediction models based on transcriptomics and machine learning.

**Methods:**

Utilizing weighted correlation network analysis (WGCNA) and random forest algorithms in GSE137570, three core gene sets of different sizes were constructed, which were externally validated in GSE66494 and GSE180394, and evaluated for their predictive performance in GSE45980 by receiver operating characteristic (ROC) curves. Predictive models were built using Cox regression, LASSO regression, and logistic regression in GSE60861. And the reliability of human CKD transcriptomic analysis and the feasibility of functional studies were validated in a mouse UUO model.

**Results:**

Combining WGCNA and differential gene analysis, 9 genes positively associated with CKD occurrence and development and 20 genes negatively associated with that were identified. By random forest algorithm, three gene sets were constructed: minimal gene set (*CCL2*, *SUCLG1*, *ACADM*), medium gene set (*CCL2*, *GGT6*, *PCK2*, *SFXN2*, *SLC34A3*, *ALPL*, *GLTPD2*, *ACADM*, *SUCLG1*), and maximal gene set (*CCL2*, *MMP7*, *GGT6*, *PCK2*, *SFXN2*, *SLC34A3*, *ALPL*, *GLTPD2*, *ACADM*, *SUCLG1*). In external validation, the maximal plage score had best classification performance for CKD (AUC:0.767) in GSE66494 and in GSE180394 (AUC:0.760), the medium plage score achieved a predictive performance for CKD progression (AUC = 0.758) in GSE45980. In the multivariate model, Cox regression analysis constructed a risk model with only minimal z-score, further LASSO regression analysis included gender and minimal z-score, but logistic regression multivariate analysis failed to be constructed with any score. A high degree of similarity between mouse CKD and human CKD in KEGG enrichment was observed in the mouse unilateral ureteral obstruction model, and the core genes related to the occurrence and progression of human CKD remained diagnostically valuable in mice.

**Conclusion:**

This study provides a transcriptomics-based risk prediction model for the occurrence and development of CKD based on machine learning, offering potential target genes for the further experimental research of CKD.

## Introduction

Chronic kidney disease (CKD) represents a significant global public health challenge, with an estimated prevalence of 14.3% (Ene-Iordache et al., 2016), affecting approximately 697.5 million individuals worldwide in 2017 (Bikbov et al., 2020). Progression to end-stage renal disease (ESRD), also known as uremia, necessitates renal replacement therapies such as dialysis or kidney transplantation. Early diagnosis and intervention are crucial for effective CKD management; however, for diagnosed patients, accurately predicting disease progression is paramount for personalized medical care and efficient allocation of healthcare resources.

Current progression risk prediction models, such as the Z6 model (Zacharias et al., 2022) based on serum markers and the PROGRESS-CKD system (Bellocchio et al., 2021) integrating renal function indicators and medical history, lack critical molecular-level information. This limitation hinders the models’ ability to identify potential biomarkers. Yuan et al. built a five-gene nomogram model for diagnosing kidney fibrosis with an AUC area of 0.923 (Yuan et al., 2025). And Lai et al. only screened out three risk hub genes associated with CKD progression including *CCR7*, *CCL21* and *CCL19* without further construction of predictive models (Lai et al., 2024). Multi-omics studies based on genomics (Galuska et al., 2024), proteomics (Schanstra et al., 2015), transcriptomics (Zheng et al., 2025), and metabolomics (Rhee et al., 2013) can significantly contribute to elucidating disease mechanisms, identifying new biomarkers and facilitating the development of more effective diagnostic and prognostic tools for CKD.

The application of machine learning algorithms to analyze multi-omics data for constructing diagnostic and prognostic models represents a burgeoning research area (Dashtban et al., 2023), given its capacity to handle high-dimensional datasets. Despite the advantages of machine learning, existing research has not fully explored the potential of integrating CKD transcriptomics data with machine learning techniques for prognostic prediction. This study aims to construct diagnostic and prognostic models for CKD using machine learning algorithms. Furthermore, we seek to explore the feasibility of functional research focusing on key risk molecules in experimental mouse models. Investigating the feasibility of functional research is essential for translating our findings into clinically relevant applications and improved patient outcomes.

## Materials and methods

### Data source

In GEO datasets, the main parameters were CKD, series, *Homo sapiens*, and tissue. From these, three datasets with larger sample sizes were selected: GSE137570 (n = 41), GSE66494 (n = 61), and GSE180394 (n = 59). Additionally, using DKD as the keyword, the dataset GSE60861 (n = 72) was chosen, which includes prognostic information. GSE60861 was reported to be divided into two cohorts with different size of microarray data including GSE45980 (n = 43) and GSE60860 (n = 29) (Rudnicki et al., 2016). All microarray datasets were normalized before further analysis.

### Differentially expressed genes and enrichment analyses

Differential analysis of the raw counts matrix was performed using the “DESeq2” package, following the standard workflow. The raw counts matrix was normalized using the VST (Variance Stabilizing Transformations) method. The differential analysis results were visualized using the R package “ggplot2”, with a log (Fold change) threshold of 1 and a *P* value threshold of 0.05. Specially, enrichment analysis for GSE60861 was conducted using DAVID (Sherman et al., 2022) and KOBAS (Bu et al., 2021).

### Weighted correlation network analysis

WGCNA analysis was conducted online using BIC (Chen et al., 2024). The soft threshold was *β* = 0.85, and the input data consisted of standardized whole-genome transcriptomes or differential gene transcriptomes. For cohort 1 of GSE137570, the selected clinical traits were Gender, GFP, TIF (degree of renal tubulointerstitial fibrosis, %), and CKD staging. For cohort 2, the input trait was CKD progression (0 = stable, 1 = progressive). The main outcomes included sample cluster, module dendrogram, module-traits correlation, and module gene-trait correlation.

### Random forest

Using the R package randomForest to perform a Random Forest analysis on the uploaded data, and visualize them using ggplot2. The importance of each variable in the classification tree is evaluated based on the Mean Decrease Gini criterion. Generally, a higher value indicates greater importance of the variable.

### Gene set scoring

The scoring methods for customized gene sets primarily include GSVA, ssGSEA, z-score, and plage. These scores are mainly generated by the “Gene Set Analysis” component of the Lianchuan Bio Cloud Platform (Lyu et al., 2023).

### Cox proportional hazard model

The analysis of prognostic data is performed using the R package “survival” (version 3.3.1) for testing the proportional hazards assumption and conducting Cox regression analysis. A prerequisite for applying Cox regression is that the covariates must satisfy the proportional hazards assumption (*P* > 0.05). The Variance Inflation Factor (VIF) can be used to assess multicollinearity among variables in the model; generally, a VIF value between 0 and 10 indicates no significant multicollinearity. Variables are included in the multivariate Cox regression analysis if their P-values meet the threshold (*P* = 0.1).

### Least absolute shrinkage and selection operator (LASSO) regression model

The cleaned data were analyzed using the R package “glmnet” (version 4.1.7) to obtain the lambda value, maximum likelihood number, or C-index, and to visualize the data. Ten-fold cross-validation was employed to screen the LASSO prognostic risk coefficients. The optimal lambda (penalty value) is referred to as lambda. min, and the lambda value within one standard error of the optimal value is referred to as lambda.1se. Additionally, the LASSO variable trajectories were observed to track the changes in the coefficients of variables entering the model. Combining non-zero parameters and variable trajectories, appropriate variables are selected.

### Logistic regression model

The glm function is used for logistic regression analysis. When the *P* value from univariate analysis meets the threshold (*P* < 0.1), the variable is included in the multivariate logistic analysis.

### Unilateral ureteral obstruction (UUO)

Male 6–8 weeks C57BL6/J mice were used to establish the UUO model. After intraperitoneal anesthesia, the mice were fixed and a laparotomy was performed to expose the surgical field using a retractor. The left ureter of the mice was ligated at both the renal and bladder ends and then transected in between. The mice were divided into the UUO group (n = 6) and the Sham group (n = 6). On postoperative day 7, tissues and organs were harvested from the mice for further study.

### Quantitative PCR

The mouse kidneys were homogenized in Trizol, and the target gene transcription levels were quantitatively analyzed through two steps: reverse transcription and real-time quantitative PCR.

The relative expression values of the target genes in the samples were calculated using the 2^−ΔΔCT^ method, and the fold change was normalized to the average expression level of the Sham group. The primers used in this study are as follows: mouse *Gapdh* forward: 5′-AGTGTTTCCTCGTCCCGTAG-3′, mouse *Gapdh* reverse: 5′-GCCGTGAGTGGAGTCATACT-3′, mouse *Fn1* forward: 5′-CCCTATCTCTGATACCGTTGTCC-3′, mouse *Fn1* reverse: 5′-TGCCGCAACTACTGTGATTCGG-3′, mouse *Col1a1* forward: 5′-CCTCAGGGTATTGCTGGACAAC-3′, mouse *Col1a1* reverse: 5′-CAGAAGGACCTTGTTTGCCAGG-3′, mouse *Ccl2* forward: 5′-GCTACAAGAGGATCACCAGCAG-3′, mouse *Ccl2* reverse: 5′-GTCTGGACCCATTCCTTCTTGG-3’.

### RNA sequencing

On postoperative day 7, total RNA was extracted from the entire kidney tissue and sent to Shenzhen Huaplo Biotechnology Co., Ltd. for sequencing analysis. After standard procedures, a transcriptome profile was generated, and subsequent analyses were performed using R.

### Statistical analysis

Bioinformatics analysis was primarily conducted using R (version 4.2.1), with statistical analyses performed using GraphPad Prism (version 8.0.2). For comparisons of means between two groups, Student’s t-test was used for statistical testing. Spearman’s correlation was employed to analyze the correlations between variables. Kaplan-Meier survival curves were constructed and compared using the log-rank test. Graphs were created using Origin 2024. A p-value <0.05 was considered statistically significant.

## Results

### Differentially expressed genes and enrichment analyses

**FIGURE 1:**
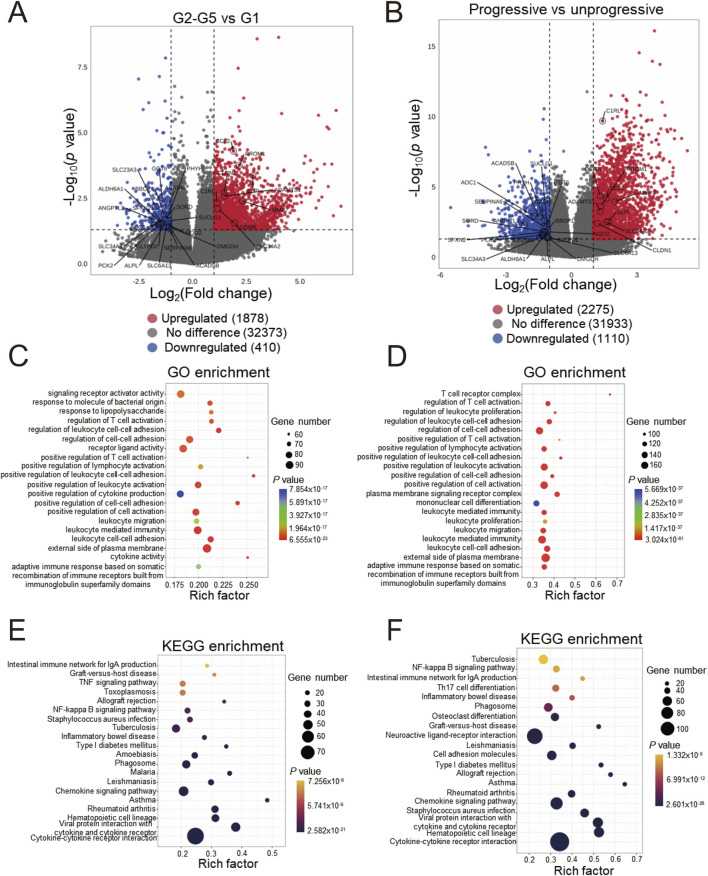
Differentially expressed genes screening and enrichment analyses of GSE137570 **(A)** Volcano plots of the differentially expressed genes (DEGs) in cohorts 1 and 2 **(B)**, the 29 most significant genes about CKD occurrence and progression are labeled; **(C)** Gene ontology (GO) enrichment analysis of cohort 1 and 2 DEGs **(D)**, along with the top 20 most significant terms, are presented; **(E)** KEGG pathway enrichment analysis of cohort 1 and 2 DEGs **(F)** and the top 20 most significant KEGG pathways are shown.

We initially employed the chronic kidney disease (CKD) dataset GSE137570 to perform differential gene screening and enrichment analysis on large-scale human kidney RNA sequencing or microarray datasets. This dataset comprises two subsets characterized by distinct attributes: one encompassing relevant clinical and pathological information (e.g., tubulointerstitial fibrosis, TIF and glomerular filtration rate, GFR) and the other reflecting CKD progression. Given the challenges in obtaining normal kidney specimens, as acknowledged in the original dataset description, the healthy control group (G1) consisted of only three individuals, while the CKD groups (G2-G5) included 21 patients. Comparative analyses between CKD cases (G2-G5, n = 21) and healthy controls (G1, n = 3) using |logFC| > 1 and *P* < 0.05 thresholds identified 1,878 upregulated and 410 downregulated genes (Figure 1A). The transcriptional profile revealed that in progressive cases (n = 8) compared to non-progressive cases (n = 9), 2,275 upregulated and 1,110 downregulated transcripts were identified (Figure 1B). Subsequently, functional enrichment analysis using Gene Ontology (GO) was performed on the differentially expressed genes. Figure 1C demonstrates that fibrosis-related functions, such as regulation of cell-cell adhesion and positive regulation of cell-cell adhesion, alongside multiple immune-related functions, including regulation of T cell activation and positive regulation of cytokine production, were enriched in CKD cases. These processes are known hallmarks of renal fibrogenesis. A similar enrichment pattern was observed in progressive CKD cases (Figure 1D). Notably, pathway analysis identified significant enrichment of immune-related pathways, such as cytokine-cytokine receptor interaction and the chemokine signaling pathway, as well as inflammatory disease pathways like graft-versus-host disease and asthma (Figure 1E,F). These pathway enrichment patterns suggest that sustained immune activation may drive both initial pathogenesis and subsequent deterioration in CKD. Collectively, the enrichment of differentially expressed genes indicates that renal immune responses are closely associated with the onset and progression of CKD.

### Identification of CKD traits-related modules by WGCNA

**FIGURE 2:**
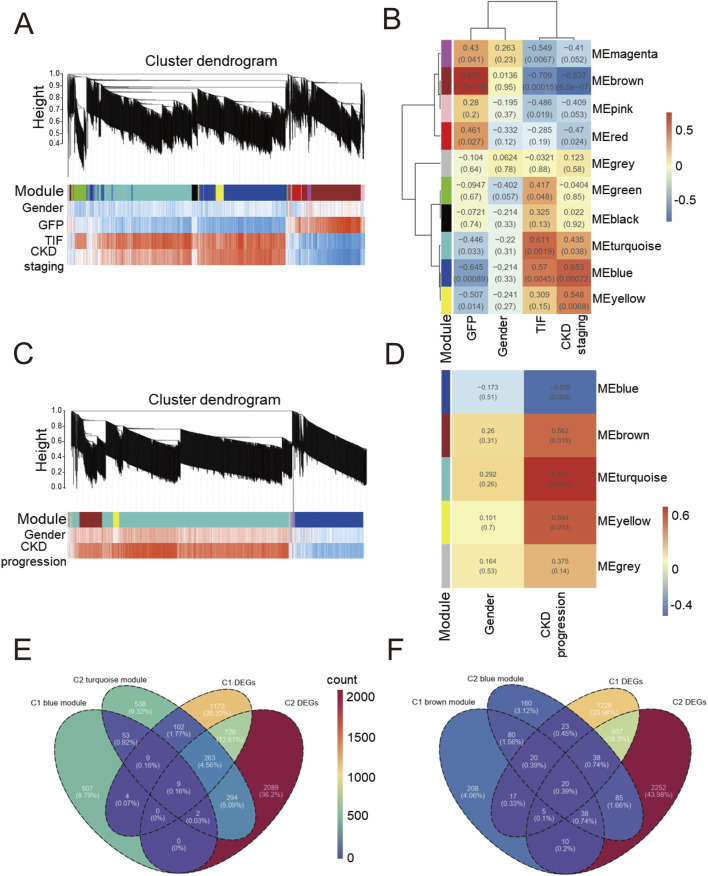
Weighted correlation network analysis of GSE137570 **(A)** Cluster dendrogram of WGCNA module plot with traits in Cohort 1 (C1); **(B)** Module-trait association heatmap with statistical significance (*P* values) in C1; **(C)** Cluster dendrogram of WGCNA module plot with traits in Cohort 2 (C2); **(D)** Module-trait association heatmap with statistical significance (*P* values) in C2; **(E)** Venn diagram of blue module in C1, differentially expressed genes (DEGs) in C1, turquoise module in C2, and DEGs in C2; **(F)** Venn diagram of brown module in C1, DEGs in C1, blue module in C2, and DEGs in C2.

We analyzed the whole genome using WGCNA to preserve transcriptional integrity and construct gene modules closely related to gender, glomerular filtration rate (GFP), tubulointerstitial fibrosis (TIF), and CKD staging. As shown in Figure 2A, genes in cohort 1 were primarily divided into 10 modules. Among these, module brown was positively correlated with GFP and negatively correlated with TIF ratio and CKD staging; conversely, module blue and module turquoise were positively correlated with TIF ratio and CKD staging and negatively correlated with GFP. However, no modules were notably associated with gender. In conjunction with Figure 2B, module brown exhibited the most notable negative correlation with CKD (*r* = −0.507, *P* = 0.014), while module blue showed the most notable positive correlation with CKD (TIF: *r* = 0.57, *P* = 0.0045; CKD staging: *r* = 0.653, *P* = 0.0068). Supplementary Figure 2A shows the correlations between genes in module blue and module brown and the traits. During sample clustering of cohort 1 (Supplementary Figure 1A), one sample, S5, was initially identified as a potential outlier. However, considering the authenticity and scarcity of this sample, and given the acceptable data quality indicated by the sample abundance distribution (Supplementary Figure 1C) and soft threshold selection curves (soft power = 16) (Supplementary Figure 1D), we included it in the analysis.

In contrast to cohort 1, WGCNA divided the entire genome spectrum of cohort 2 into 5 modules. As shown in Figures 2C,D, module turquoise had the most notable positive correlation with CKD progression (*r* = 0.701, *P* = 0.0017), while module blue had the most notable negative correlation (*r* = −0.532, *P* = 0.028). Supplementary Figure 2B shows the correlations between genes in module turquoise and the traits. Similar to cohort 1, no modules were found to be notably associated with gender, and no outliers were detected in the sample clustering of cohort 2 (Supplementary Figure 1B). Given that module blue in cohort 1 and module turquoise in cohort 2 both demonstrated a positive correlation with CKD and CKD progression, respectively, we sought to identify commonly dysregulated genes. As shown in Figures 2E,F, we intersected the differentially expressed genes from these two modules. In dataset GSE137570, we identified 9 genes that were notably positively correlated with CKD development, defining them as the positive gene set; similarly, a negative gene set consisting of 20 genes was notably negatively correlated with CKD development. Through WGCNA, we initially screened out a risk gene set for CKD and a protective gene set for CKD.

### Identification of CKD-related core genes and core gene sets

**FIGURE 3:**
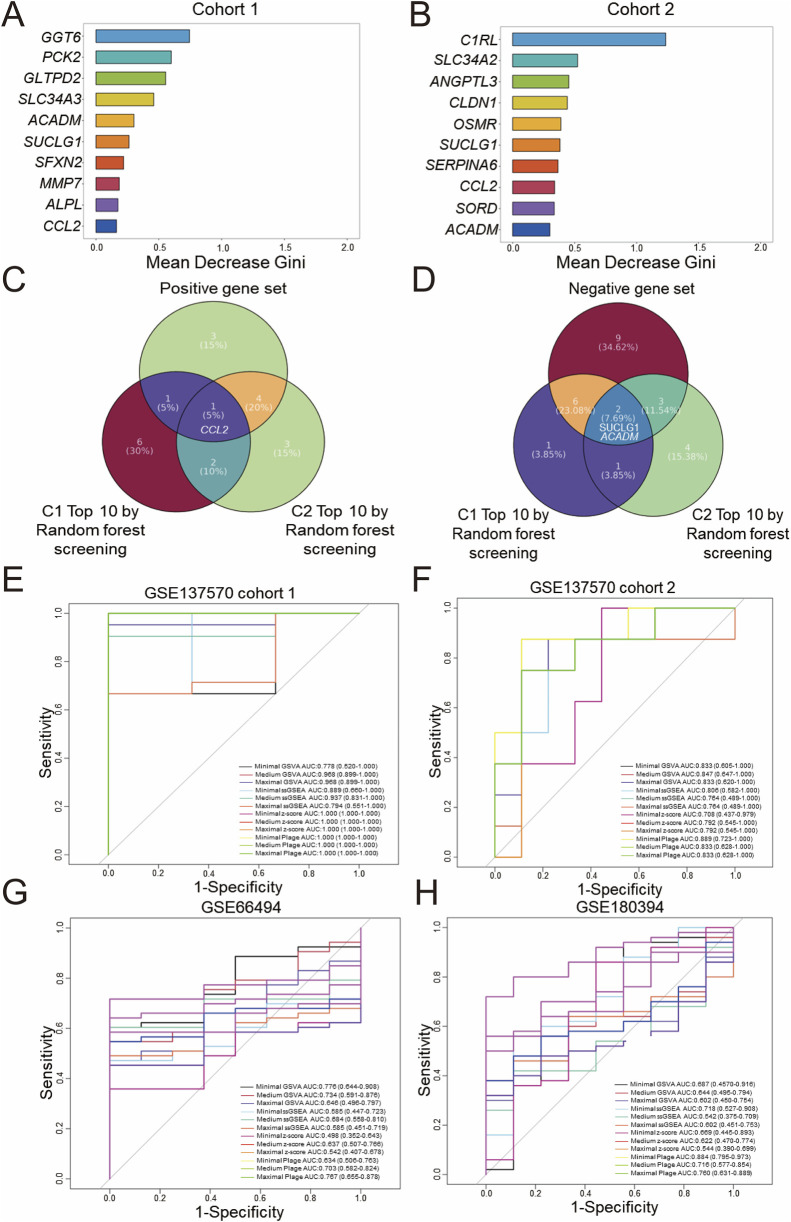
Screening and diagnostic capacity of core genes. Random forest screening based on the Gini index was performed on the GSE137570 dataset, cohort 1 **(A)** and cohort 2 **(B)**, and the top 10 genes with the highest mean decrease in Gini index are listed. **(C)** A Venn diagram showing the overlap between positive and negative gene sets **(D)**, classified in Figures 2E,F, and the top 10 genes identified in **(A,B)**. The diagnostic performance of three predefined gene sets evaluated by four scoring algorithms in the discovery dataset (GSE137570 cohort 1, cohort 2) **(E,F)** was assessed and then in validation datasets included GSE66494 (controls n = 8 vs. CKD n = 53) **(G)** and GSE180394, a validation dataset containing healthy (n = 9) and etiologically diverse CKD samples (n = 50) **(H)**.

To further identify core genes related to CKD, the random forest algorithm was employed to rank the DEGs in Cohort 1 by importance. Displayed in Figure 3A are the top 10 genes most strongly associated with CKD onset based on this ranking. Presented in Figure 3B are the top 10 genes most strongly associated with CKD progression in Cohort 2. Subsequently, as shown in Figure 3C, intersecting the positive gene set with the top 10 genes from the random forest analysis of Cohort 1 identified *CCL2* as a core positively correlated gene. Following this, as displayed in Figure 3D, two core negatively correlated genes, *SUCLG1* and *ACADM*, were identified. A Minimal gene set (*CCL2*, *SUCLG1*, *ACADM*) was then constructed.

Drawing upon previous literature that utilized DEGs to divide genes into modules (39402203), an attempt was made to reconstruct WGCNA analysis based on DEGs in both Cohort 1 and Cohort 2 of GSE137570. As presented in Supplementary Figure 3A,B, the DEGs in Cohort 1 were divided into 4 gene modules, with the blue module (*r* = 0.759, *P* < 0.0001) demonstrating the most significant positive correlation with CKD onset, and the brown module (*r* = −0.815, *P* < 0.0001) demonstrating the most significant negative correlation. In Cohort 2, the turquoise module (*r* = 0.75, *P* = 0.00053) exhibited the most significant positive correlation with CKD progression, while the grey module (*r* = −0.869, *P* < 0.0001) exhibited the most significant negative correlation. Compared to Figures 2B,D, the gene modules constructed by DEGs in WGCNA analysis exhibited higher correlations with fewer modules.

Next, as shown in Supplementary Figure 3C,D, the most significant module genes were intersected with the top 10 important genes screened by random forest. It was observed that the core genes most significantly positively correlated with CKD onset were *CCL2* and *MMP7*, while the core genes most significantly negatively correlated were *GGT6*, *PCK2*, *SFXN2*, *SLC34A3*, *ALPL*, *GLTPD2*, *ACADM*, and *SUCLG1*. The core genes most significantly positively correlated with CKD progression were *CCL2*, *CLDN1*, *SLC34A2*, *OSMR*, and *C1RL*; however, no core genes were identified as most significantly negatively correlated with CKD development. Considering that a gene set composed of approximately 10 core genes offers improved clinical translational feasibility, a maximal gene set (*CCL2*, *MMP7*, *GGT6*, *PCK2*, *SFXN2*, *SLC34A3*, *ALPL*, *GLTPD2*, *ACADM*, *SUCLG1*) and a medium gene set (*CCL2*, *GGT6*, *PCK2*, *SFXN2*, *SLC34A3*, *ALPL*, *GLTPD2*, *ACADM*, *SUCLG1*) were constructed for subsequent analyses. By integrating WGCNA and random forest analyses of two gene expression profiles (whole-genome and DEGs), three gene sets of varying sizes related to CKD onset and progression were screened.

The aforementioned three gene sets were scored using different gene set enrichment algorithms, including GSVA, ssGSEA, z-score, and PLAGE, and the classification accuracy of these scores was further evaluated using ROC curves for binary diagnosis. For internal validation in Cohort 1 of GSE137570, as presented in Figure 3E, all scoring methods, with the exception of minimal GSVA (AUC: 0.778) and maximal ssGSEA (AUC: 0.794), which demonstrated moderate diagnostic performance with AUC <0.8, exhibited excellent diagnostic performance (AUC >0.8). Displayed in Figure 3F, internal validation in Cohort 2 indicated that all scoring methods except medium ssGSEA (AUC: 0.764), maximal ssGSEA (AUC: 0.764), minimal z-score (AUC: 0.708), medium z-score (AUC: 0.792), and maximal z-score (AUC: 0.792), which demonstrated moderate performance, had excellent diagnostic performance.

Subsequently, GSE66494 (n = 61, whole-kidney array profiling) and GSE180394 (n = 59, renal tubule array profiling) were used as external validation datasets to assess the diagnostic performance of different scoring methods and gene set combinations. These datasets were selected to represent different aspects of CKD pathology, with GSE66494 providing a broad assessment of the whole kidney and GSE180394 focusing specifically on the renal tubules. As displayed in Figure 3G, in the diagnostic validation of GSE66494, only minimal GSVA (AUC: 0.776), medium GSVA (AUC: 0.734), medium PLAGE (AUC: 0.703), and maximal PLAGE (AUC: 0.767) demonstrated moderate diagnostic performance. Validation using GSE180394, as shown in Figure 3H, indicated that minimal ssGSEA (AUC: 0.718), medium PLAGE (AUC: 0.716), and maximal PLAGE (AUC: 0.760) exhibited moderate diagnostic performance for CKD, while minimal PLAGE (AUC: 0.884) exhibited excellent diagnostic performance. In summary, based on the evaluation of the diagnostic performance of different gene sets and scoring methods, standardization of a single gene set and a single scoring method in this study remains challenging, and further integration with prognostic diagnostic models.

### Construction of prognostic prediction model

GSE60861 is a CKD dataset encompassing comprehensive clinical test data and prognostic information, with the observation endpoint defined as progression to end-stage renal disease or a doubling of serum creatinine (Cr) (Rudnicki et al., 2016).

**FIGURE 4:**
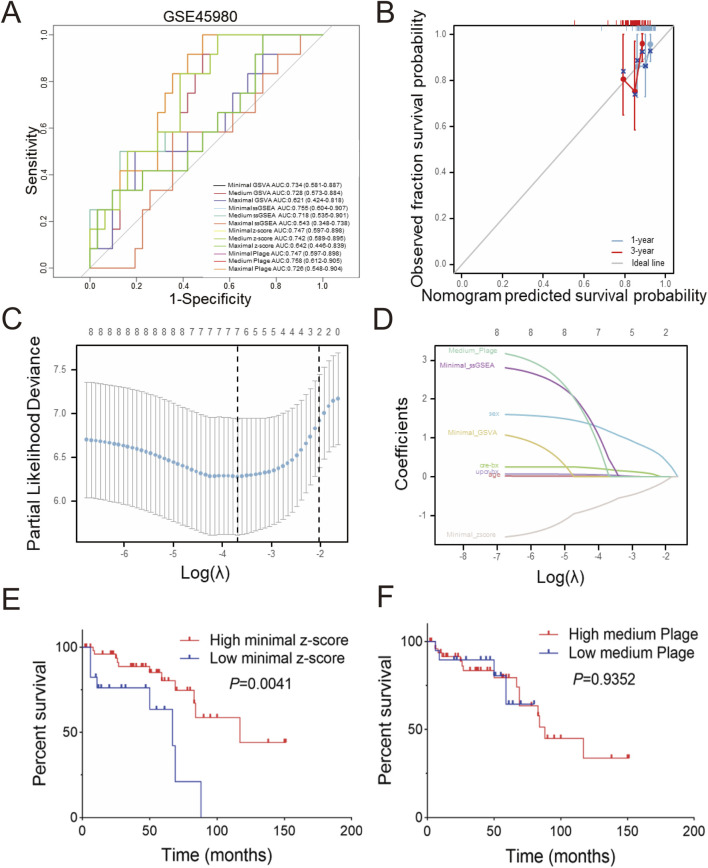
Predictive and diagnostic value of minimal z-score in GSE60861 **(A)** Total twelve predictive values of different scoring in GSE45890, **(B)** Validation of medium plage score calibration curve in GSE60861 during one or 3 years observation window, **(C)** LASSO coefficient paths of GSE60861, **(D)** LASSO variable trajectory diagram, **(E)** Kaplan-Meier curve of progression-free survival based on minimal z-score in GSE60861, with the cutoff at the first quartile, **(F)** Kaplan-Meier curve of progression-free survival based on the medium plage score in GSE60861, with the cutoff at the first quartile.

Diagnostic receiver operating characteristic (ROC) assessments were conducted on the GSE60861 dataset including GSE45980 and GSE60860 cohorts. As illustrated in Figures 4A,A few scoring methods achieved a moderate but not excellent level of accuracy in predicting CKD progression within the GSE45980 dataset, with the medium plage score demonstrating the best performance (AUC: 0.758). Calibration of the prediction based on the medium plage score (Figure 4B) indicated acceptable accuracy for predicting CKD progression within one or 3 years.

Univariate and multivariate Cox regression analyses were performed to identify risk factors associated with CKD progression. Table 1 summarizes the results of univariate Cox regression analysis. Females exhibited a lower risk of CKD progression compared to males (HR: 0.159, *P* = 0.004), while age (HR: 1.031, *P* = 0.047) and serum creatinine-BX (time of biopsy) (HR: 1.817, *P* < 0.001) were identified as risk factors. Among the four candidate scoring algorithms, only minimal z-score (HR: 0.494, *P* = 0.005) was significantly associated with lower risk of CKD progression. Using *P* < 0.1 as the significance threshold for inclusion in the multivariate Cox regression analysis, the table indicated that minimal z-score (HR: 0.226, *P* = 0.018) was independent low-risk factor for CKD progression, while gender, age and creatinine-BX were not included. These findings suggest the potential of gender, age, creatinine-BX, and minimal z-score to form a multivariate prognostic prediction model.

**TABLE 1:** Univariate and multivariate Cox regression analysis of GSE60861.

Characteristics	Total(N)	Univariate analysis		Multivariate analysis	
		Hazard ratio (95% CI)	P value	Hazard ratio (95% CI)	P value
Sex	72				
Male	43	Reference		Reference	
Female	29	0.159 (0.046–0.554)	**0.004**	0.282 (0.077–1.037)	0.057
Age (years)	72	1.031 (1.000–1.062)	**0.047**	1.017 (0.982–1.053)	0.346
Creatinine-BX (mg/dl)	72	1.817 (1.310–2.521)	**<0.001**	1.338 (0.874–2.048)	0.181
UPCR-BX (g/g)	70	1.027 (0.894–1.180)	0.704		
Minimal GSVA	72	0.362 (0.130–1.010)	0.052	7.049 (0.510–97.518)	0.145
Minimal ssGSEA	72	0.280 (0.056–1.407)	0.122		
Medium Plage	72	6.298 (0.384–103.265)	0.197		
Minimal z-score	72	0.494 (0.303–0.807)	**0.005**	0.226 (0.066–0.772)	**0.018**

Bold values in the table represent statistically significant values.

Furthermore, LASSO regression analysis was employed to screen the aforementioned potential predictors, including gender, age, creatinine-BX, UPCR-BX and four scores with best performance in Figure 4A (minimal GSVA, minimal ssGSEA, minimal z-score, and medium plage). As shown in Figure 4C, after selecting the optimal lambda.1se (Log(λ) = -2), four non-zero parameters were selected: gender (coefficient: 0.436) and minimal z-score (coefficient: 0.111). Integrating this with Figure 4D revealed that gender and minimal z-score contributed substantially to the prediction model, while the contribution of age and UPCR-BX was comparatively stable.

In internal validation, to evaluate the predictive performance of the Cox regression and LASSO regression models for CKD progression, Cox risk scores were stratified into groups based on the median. Supplementary Figure 4A demonstrates that the High Cox risk score group exhibited a shorter progression-free survival period (*P* = 0.0012). Meanwhile, Supplementary Figure 4B shows that the high LASSO risk score group showed a shorter free-progression survival (*P* = 0.003).

Univariate and multivariate logistic regression models were also used to assess the predictive performance of the factors. As shown in Table 2, none factor was identified as an independent risk factor for CKD progression. Only in the univariate logistic regression analysis were gender (univariate OR: 0.176, *P* = 0.011; multivariate OR: 0.270, *P* = 0.074), creatinine-BX (univariate OR: 1.721, *P* = 0.020; multivariate OR: 1.395, *P* = 0.182) and minimal z-score (univariate OR: 0.555, *P* = 0.022; multivariate OR: 0.195, *P* = 0.079) predictive of CKD progression risk.

**TABLE 2:** Univariate and multivariate Logistic regression analysis of GSE60861.

Characteristics	Total(N)	Univariate analysis		Multivariate analysis	
		Odds Ratio (95% CI)	P value	Odds Ratio (95% CI)	P value
Gender	72				
Male	43	Reference		Reference	
Female	29	0.176 (0.046–0.676)	**0.011**	0.270 (0.064–1.134)	0.074
Age (years)	72	1.024 (0.993–1.056)	0.131		
Creatinine-BX (mg/dl)	72	1.721 (1.091–2.715)	**0.020**	1.395 (0.855–2.274)	0.182
UPCR-BX (g/g)	70	1.041 (0.892–1.216)	0.609		
Minimal GSVA	72	0.390 (0.134–1.136)	0.084	12.726 (0.311–520.136)	0.179
Minimal ssGSEA	72	0.281 (0.045–1.750)	0.174		
Medium Plage	72	5.401 (0.245–119.033)	0.285		
Minimal z-score	72	0.555 (0.336–0.917)	**0.022**	0.195 (0.031–1.210)	0.079

Bold values in the table represent statistically significant values.

The relationship between the minimal z-score or medium plage scoring methods and CKD progression was further investigated through survival analysis. Figure 4E indicates that the high minimal z-score group had a longer progression-free survival time (*P* = 0.0041). But, Figure 4F demonstrates that there was no significance between two medium plage group (*P* = 0.9352). In summary, we developed a Cox risk prediction model for CKD using the minimal z-score and established a LASSO regression prediction model for CKD incorporating both gender and minimal z-score.

### Validation of bioinformatics results

**FIGURE 5:**
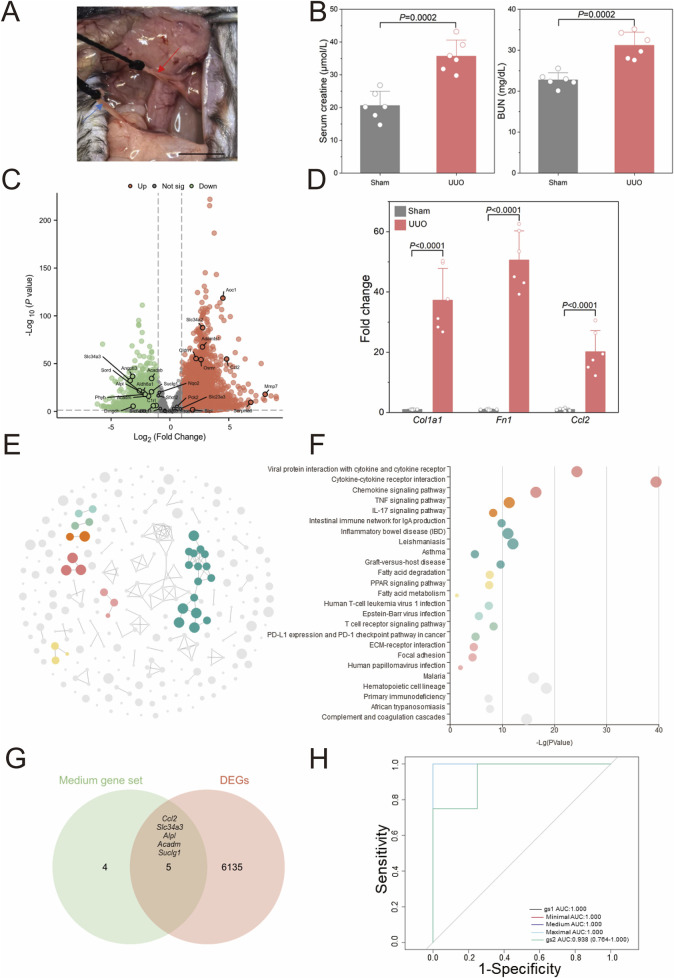
Validation of human CKD bioinformatics analyses **(A)** Representative surgical views of the unilateral ureteral obstruction (UUO) model are shown. The red arrow indicates the left ureter proximal to the left kidney of the mouse, and the blue arrow indicates the distal ureter near the bladder. **(B)** Kidney function evaluated by serum creatinine and urine nitrogen level shown for UUO and Sham mice (n = 6). **(C)** The volcano plot illustrates differentially expressed genes (DEGs) identified via whole kidney RNA sequencing in the UUO group compared to the sham group (n = 4). **(D)** Validation of RNA sequencing results by quantitative polymerase chain reaction (qPCR) is shown (n = 6). **(E)** CircFunMap analysis of enriched Kyoto Encyclopedia of Genes and Genomes (KEGG) terms is presented, where each node represents an enriched term, node color represents different clusters, and node size represents the enriched *P* value. **(F)** The bubble plot displays enriched KEGG terms, with the top five terms with the highest enrichment ratio from each cluster shown. **(G)** A Venn diagram depicts the overlap between a medium 9-gene set and DEGs in the UUO model. **(H)** Receiver operating characteristic (ROC) curves for the four gene sets in diagnosing UUO are shown **(H)**, gene set 1 (gs1) represented the overlapping genes in **(G)**, gene set 2 (gs2) contained the 29 risk genes screened from GSE137570. Scale bar: 1 cm. *P* values were calculated using two-tailed Student’s t-test assuming equal variances following confirmation of normal distribution by Shapiro-Wilk test.

To validate the stability of the aforementioned bioinformatics screening and model construction, we established a unilateral ureteral obstruction (UUO) model in mice, one of common models for CKD characterized by kidney fibrosis (Chen et al., 2023), and obtained transcriptome sequencing data from the kidneys. Figure 5A presents a real operation view illustrating the left ureter ligation during UUO model induction. Seven days post-operation, kidney tissues and serum were harvested. Figure 5B demonstrates that blood creatinine (*P* = 0.0002) and urea nitrogen (*P* = 0.0002) showed significant elevation in UUO versus sham groups. Following cross-species ortholog mapping, the 29 key human CKD genes identified previously, including *Col1a1* and *Fn1*, were highlighted in the differential gene analysis (Figure 5C). Then, qPCR validation in Figure 5D reveals significant upregulation of *Col1a1* (37.32 ± 10.50 vs. 1.00 ± 0.12, *P* < 0.0001), *Fn1* (50.62 ± 9.62 vs. 1.00 ± 0.18, *P* < 0.0001), and *Ccl2* (20.18 ± 7.00 vs. 1.00 ± 0.42, *P* < 0.0001) in the UUO group comparing to sham group.

Figures 5E,F present the KEGG pathway enrichment analysis of differentially expressed genes (DEGs) between UUO and sham groups, revealing significant alterations in seven major pathways clusters following UUO induction, including immune-related pathways such as cytokine-cytokine receptor interaction and TNF signaling. Inflammation-related pathways such as inflammatory bowel disease and asthma were also significantly enriched; these pathways have been previously implicated in renal fibrosis mechanisms. The transcriptional profile of the affected kidney in the UUO model thus closely resembled the transcriptomic changes observed in human CKD. We further identified five significantly expressed core human CKD genes (*Ccl2*, *Slc34a3*, *Alpl*, *Acadm*, and *Suclg1*) from the DEGs in the mouse model (Figure 5E) and designated them as gene set 1 (gs1). The 29 initially screened genes were designated gene set 2 (gs2). Here, gs2 represents initial screening candidates while gs1 contains evolutionarily conserved core genes. Using the z-score integration algorithm, the diagnostic performance for CKD in mice showed strong diagnostic capability (AUC >0.9) regardless of the size of the custom gene sets in Figure 5H.

## Disscussion

The mechanisms underlying the development and progression of chronic kidney disease (CKD) remain incompletely elucidated. Accurate prognosis is crucial for improving patient outcomes. The development of prognostic prediction models for CKD offers a translational approach to assess patient prognosis and inform clinical decision-making.

In this study, bioinformatics, Weighted Gene Co-expression Network Analysis (WGCNA), and random forest algorithms were employed to identify molecules associated with CKD onset and progression at the transcriptional level using the human dataset GSE137570. To evaluate the predictive performance of gene sets, a three-stage filtering process was implemented: first, Cox regression was applied to establish a baseline model; second, LASSO regression was used for feature reduction; and finally, logistic regression was performed to clarify the risk factors. The effectiveness of the minimal z-score, which is associated with CKD progression, was validated. The reliability of the bioinformatics analysis of the human dataset was subsequently confirmed in a mouse unilateral ureteral obstruction (UUO) model, demonstrating the feasibility of in-depth mechanistic studies using this model.

This study identified a core gene set of nine genes and a minimal functional gene set of three genes significantly associated with the onset and progression of CKD based on transcriptomic analysis and risk model construction. The feasibility of studying these biomarkers in a mouse UUO model was also demonstrated.

Machine learning methods have become increasingly prevalent in the prognosis of kidney diseases (Sanmarchi et al., 2023). While this study successfully constructed CKD prediction models using various regression techniques, the prognostic effectiveness requires further improvement. Several limitations inherent to transcriptomic risk models warrant consideration. Firstly, the number of CKD datasets with prognostic information is limited, as is the sample size of sequenced patients. Secondly, the high biological heterogeneity among different CKD subtypes introduces complexity in constructing highly effective molecular diagnostic model.

Addressing these limitations necessitates exploring several strategies. One approach involves integrating multi-omics data to develop comprehensive molecular prognostic tools (Provenzano et al., 2022). Another strategy is combining diverse machine learning algorithms to mitigate selection bias for core risk genes. While some studies have constructed CKD progression-related prognostic models using machine learning applied to laboratory data (Ferguson et al., 2022), their evaluation metrics remain limited. Furthermore, risk prediction models based solely on traditional laboratory data often fail to elucidate the underlying molecular mechanisms driving CKD progression. A study using patient kidney transcriptomics and urinary proteomics for risk stratification (Reznichenko et al., 2024) suggests that individual omics data possess significant value for precision treatment. Therefore, future comprehensive risk models incorporating current medical history, laboratory indicators, multi-omics datasets, and machine learning algorithms hold promise for personalized treatment decisions in CKD.

This study identified three core genes—*CCL2*, *SUCLG1*, and *ACADM*—and validated similar transcriptomic changes between human CKD and the mouse UUO model, suggesting the UUO model as a platform for investigating the molecular mechanisms and functional roles of CKD biomarkers. Experimental evidence indicates that high *CCL2* expression in infiltrating macrophages and neutrophils is associated with renal fibrosis in CKD (Puthumana et al., 2021). Clinical observation reveals significantly increased *CCL2* concentrations in the plasma of human CKD patients (Schettini et al., 2022). This finding, coupled with clinical observations, suggests an important biological role for *CCL2* in CKD onset and progression, although further investigations using cell-specific knockout and other gene-editing strategies in mice are needed to elucidate the specific mechanisms. Prior research demonstrated that *SUCLG1* promotes mitochondrial biogenesis, leading to leukemia progression through POLRMT succinylation (Yan et al., 2024), indicating its role in post-translational modification. However, its role in CKD remains largely unexplored. Genome-wide association studies have identified a correlation between *ACADM* and plasma metabolic markers in CKD patients, yet the biological function of *ACADM* in CKD requires further investigation.

In conclusion, this study offers a transcriptomics-based risk prediction model for CKD onset and progression, providing potential target genes for molecular mechanism research and effective tools for exploring omics-based prognostic prediction in CKD patients.

## Conclusion

Using WGCNA and random forest algorithms, we constructed core gene sets of varying sizes from CKD-related transcriptomic datasets. The performance of different gene set scores showed differential diagnostic accuracy across datasets. To clarify the application value of these core gene sets, we developed CKD progression prediction models. Specifically, we used the core gene sets in three distinct ways: (1) a Cox proportional hazards model for survival analysis, (2) LASSO regression for feature selection, and (3) logistic regression for classification prediction, integrating key clinical indicators such as eGFR and gender. We performed cross-species validation through comparative analysis of the mouse unilateral ureteral obstruction (UUO) model and human CKD transcriptomics, demonstrating the reliability of our bioinformatics analysis of human CKD datasets and the feasibility of the mouse UUO model for functional studies of CKD-related risk genes. This study provided effective classification and progression risk prediction models for CKD diagnosis and prediction, laying a foundation for further mechanistic research into the roles of specific genes, such as *CCL2*, *SUCLG1* and *ACADM*.

## Data Availability

All public human chronic kidney disease datasets used in this article can be queried and downloaded from the GEO website by GEO number including GSE137570, GSE66494, GSE180394, GSE45980 and GSE60861. The raw data for mouse transcriptome sequencing in this article has been uploaded to Supplementary Material.

## Abbreviations

CKD, Chronic kidney disease; DEGs, Differentially expressed genes; GO, Gene ontology; KEGG, Kyoto Encyclopedia of Genes and Genomes; WGCNA, Weighted correlation network analysis; C1, cohort 1; C2, cohort 2; TCGA, The Cancer Genome Atlas.
