# Supplementary material for: Identification of progression-related genes and construction of prognostic model for chronic kidney disease by machine learning
Source: Front Cell Dev Biol. 2025 Aug 15;13:1627355. doi: 10.3389/fcell.2025.1627355 (PMC12395503; doi:10.3389/fcell.2025.1627355)
Supplement: Supplementary file 1 [file DataSheet1.docx]

**
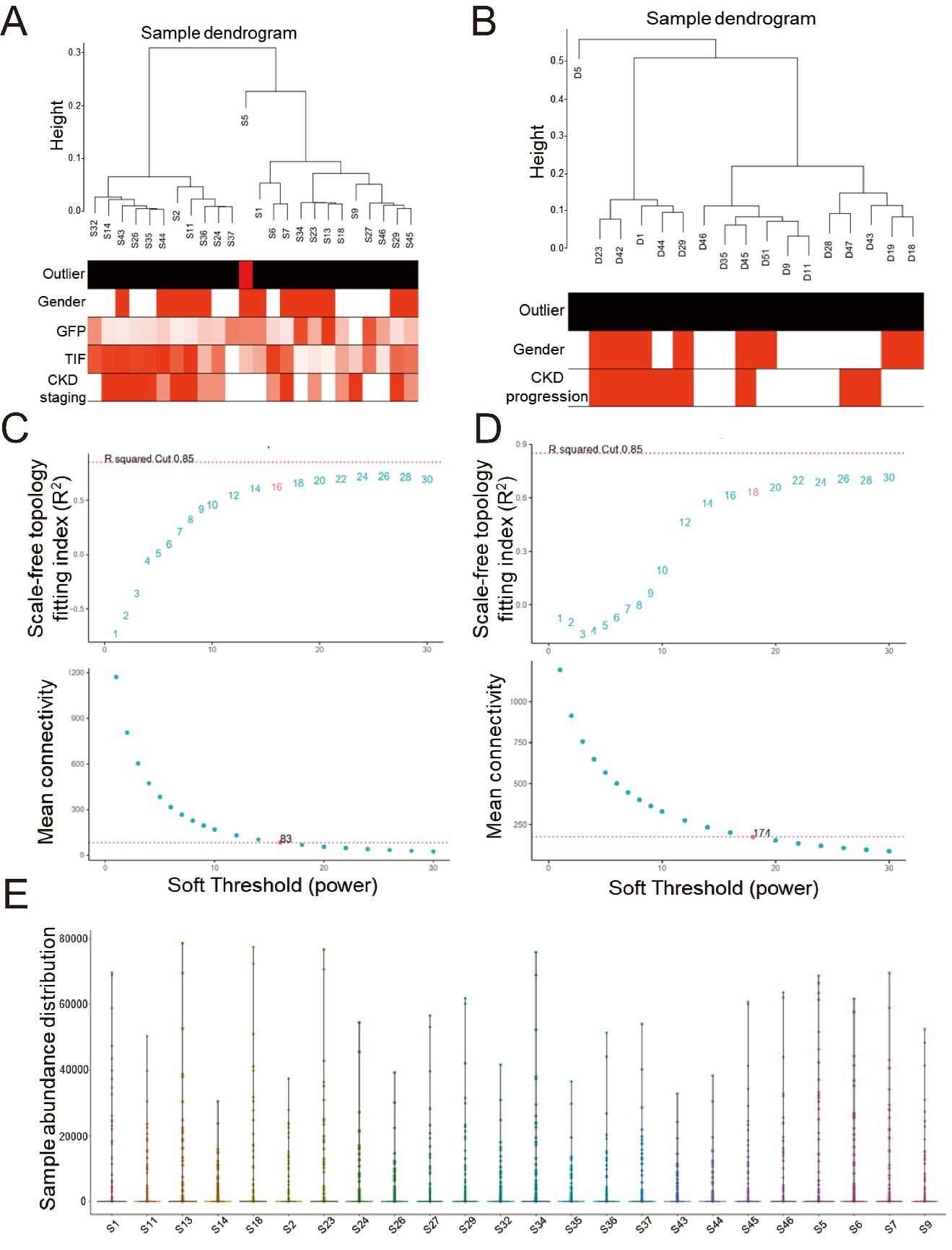
**

**Supplementary Figure 1. WGCNA of GSE137570**

（A）Outlier detection of sample cluster in cohort 1, and cohort 2 (B), (C) Soft power detection on cohort 1, and cohort 2(D) (E) Abundance profile distribution of all samples in cohort 1.


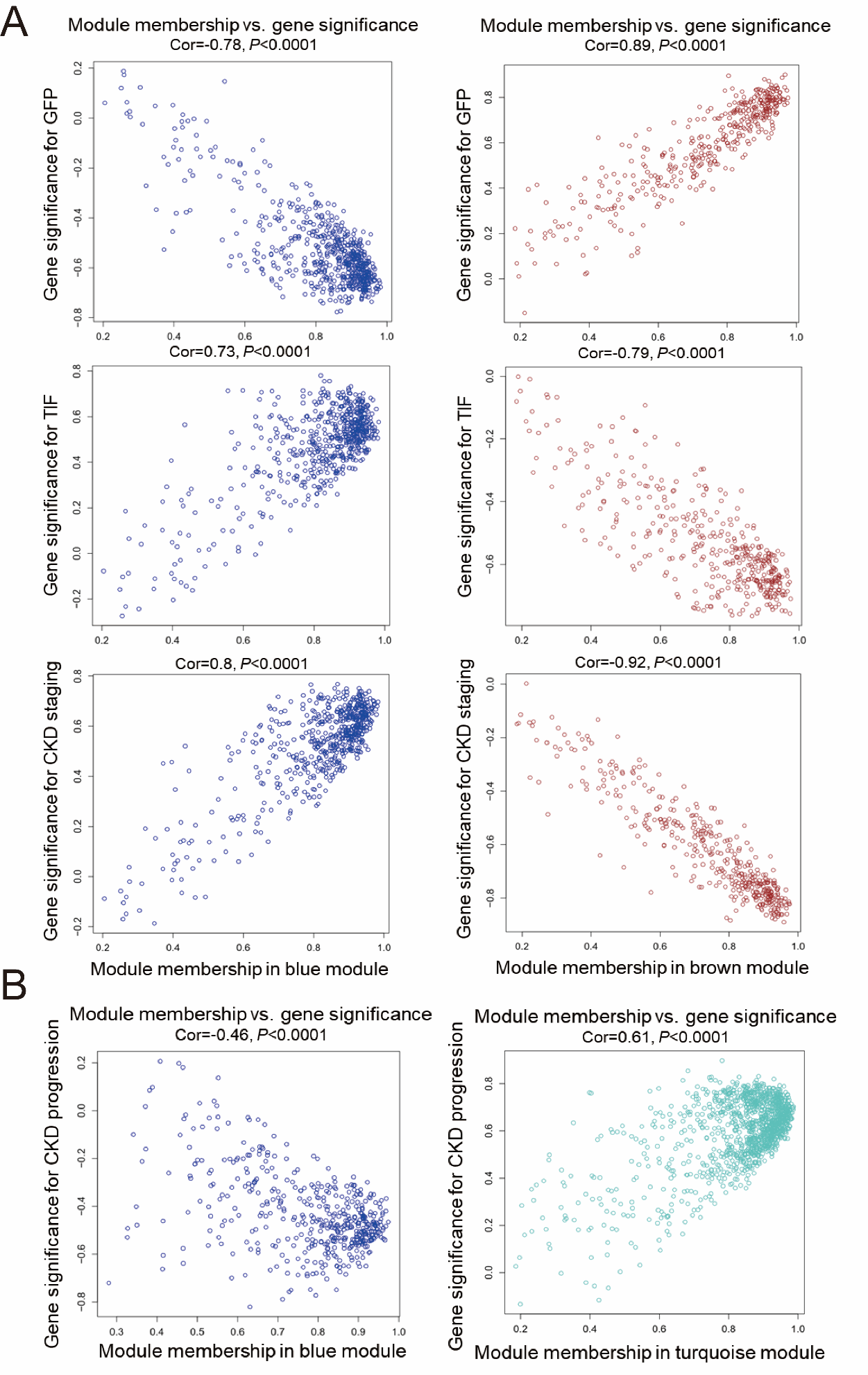


**Supplementary Figure 2. Module membership of genes in most significant modules**

(A) Genes significance for GFP, or TIF, or CKD staging in blue module and brown module, (B) Genes significance for CKD progression in blue module and turquoise module.


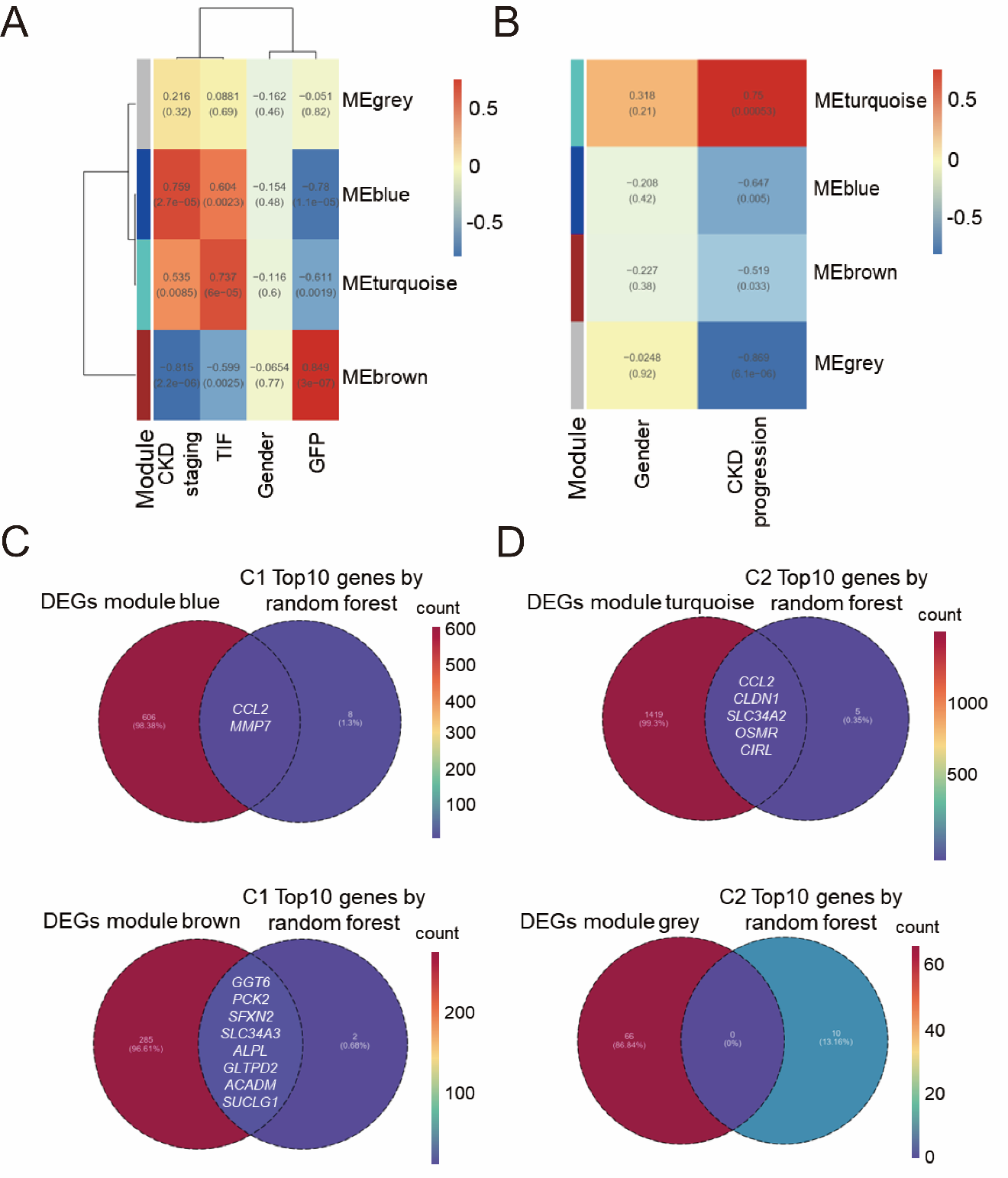


**Supplementary Figure 3. WGCNA based on DEGs of GSE137570**

(A)Module correlation plot of cohort 1, and cohort 2(B), (C) Venn plots of Cohort 1 top 10 genes by random forest with DEGs module blue or module brown. (D) Venn plots of Cohort 2 top 10 genes by random forest with DEGs module turquoise or module grey.


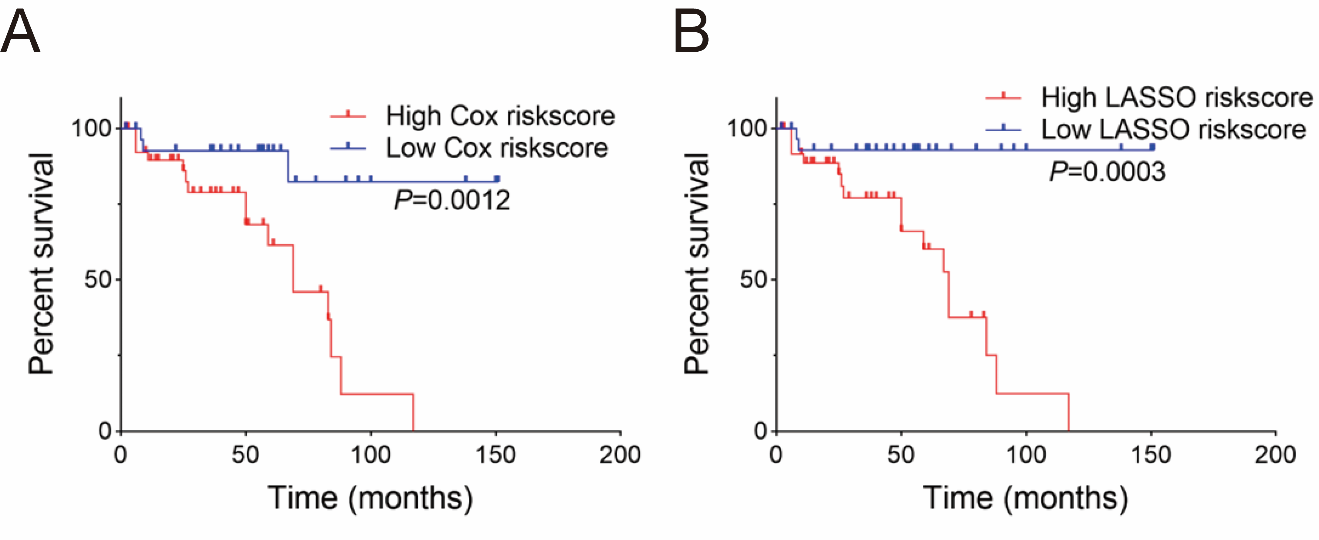


**Supplementary Figure 4. Validation of Cox regression model and LASSO regression model in GSE60861**

(A) Kaplan-Meier curve based on Cox risk score in GSE60861.Cutoff is median. (B) Kaplan-Meier curve based on Cox risk score in GSE60861.Cutoff is median.
